# Fiocruz Biological Collections: strengthening Brazil's biodiversity knowledge and scientific applications opportunities

**DOI:** 10.3897/BDJ.8.e53607

**Published:** 2020-06-24

**Authors:** Manuela da Silva, Marcia Chame, Ricardo Moratelli

**Affiliations:** 1 Vice-presidency of Research and Biological Collections, Oswaldo Cruz Foundation, Rio de Janeiro, Brazil Vice-presidency of Research and Biological Collections, Oswaldo Cruz Foundation Rio de Janeiro Brazil; 2 Institutional Platform Biodiversity and Wild Health, Oswaldo Cruz Foundation, Rio de Janeiro, Brazil Institutional Platform Biodiversity and Wild Health, Oswaldo Cruz Foundation Rio de Janeiro Brazil; 3 Fiocruz Atlantic Forest, Oswaldo Cruz Foundation, Rio de Janeiro, Brazil Fiocruz Atlantic Forest, Oswaldo Cruz Foundation Rio de Janeiro Brazil

**Keywords:** *ex situ* conservation, public health, biodiversity, biological resource centre, genetic resource

## Abstract

Biological collections are central in understanding and preserving life on Earth. In Brazil, the most representative collections are kept by natural history museums, whose primary focus is in invertebrates, vertebrates and vascular plants. Only a few institutions keep repositories in different kingdoms. The Oswaldo Cruz Foundation (Fiocruz), established in 1900, is a strategic public health institution of the Ministry of Health of Brazil. As such, Fiocruz is responsible for a wide range of activities, from basic research to the development and production of vaccines, drugs, reagents and diagnostic kits. Its biological collections were soon established in the expeditions made by naturalists and physicians seeking integrated knowledge of the fauna, flora and tropical diseases. Since then, they have been part of the institutional policy. In a few decades, those collections were already in the forefront of basic and applied research on tropical parasitic and infectious diseases. Currently, they comprise thirty-three repositories representing part of the Brazilian diversity of bacteria, fungi, protozoa, helminths, arthropods, molluscs and plants of medical and environmental importance. Different methods of long-term preservation are applied for the conservation of this wide range of organisms represented by about 6 million specimens. Herein, we describe this range of collections and discuss their complementary role as repositories of groups not represented in other biological collections in Brazil. These valuable biological materials have been used in public health and medical research, as well as for technological development and innovation in Brazil. Parallel to this specific usage, Fiocruz biological collections have played and continue to play a unique and important role in understanding and conserving part of Brazil’s biodiversity that is currently under-represented in other biological and natural history collections in Brazil and South America.

## Introduction

Biological diversity, as more broadly understood, can be defined as the variability of life on Earth, including all living organisms and the evolutionary, ecological and behavioural complexes of which they are part (Nations 1992). According to the Convention of Biological Diversity, each party shall, as far as possible, adopt measures for the *ex-situ* conservation of components of biological diversity and establish and maintain facilities for *ex-situ* conservation of plants, animals and microorganisms, preferably in the country of origin of genetic resources (Nations 1992). Biological collections are the repositories for this biological diversity and, at present, are held by culture collections, natural history museums, herbaria and other science centres (Moratelli 2014).

Therefore biological collections, as part of the *ex situ* conservation strategy, are essential parts of the research infrastructure of all countries and they are critical to many areas including conservation, scientific research and technological development. The important role of biological collections within this scope, has been recognised by the current Brazilian legislation, Law 13,123/2015, for access to genetic resources and associated traditional knowledge and benefit sharing, as well as by the European Union Regulation (EU) 511/2014, which is based on the Nagoya Protocol on Access to Genetic Resources and the Fair and Equitable Sharing of Benefits Arising from their Utilization (Davis et al. 2016).

Brazil stands out as being one of the most biodiverse places on Earth and such diversity can gain even more value when properly maintained, organised, identified, classified, documented and available for access, whenever there is demand, as occurs in the biological collections. However, the majority of repositories devoted to document biodiversity in Brazil are mainly focused on animal and plant diversity. On the other hand, Brazilian institutions primarily focused on health, agronomy, food security etc. have also created biological collections to support applied research. These collections have played a secondary (and under-appreciated) role in representing biodiversity not held in collections of other institutions devoted to document life. Hereafter, we analyse the complementary role of biological collections of the Oswaldo Cruz Foundation (Fiocruz)—a Brazilian federal institution that coordinates a well-structured network of epidemiological control and public health—in representing a portion of the biodiversity in Brazil that is not represented by collections held by other institutions.

Fiocruz hosts different biological collections whose oldest specimens come from the early 20th century. During scientific expeditions and actions to combat disease outbreak and emergence, Fiocruz researchers collected, analysed and deposited biological material from different regions of Brazil, comprising all biomes represented in the country. Rapidly, those early biological collections became part of the institutional policy already focused on fighting parasitic diseases caused by bacteria and protozoa and transmitted by insects, molluscs and other vectors (daSilva et al. 2011, daSilva and Sá 2016).

Although those early collections have their roots in epidemiological investigations, they are fundamental today for the knowledge and conservation of biodiversity. These collections have been expanded with time and new taxonomic groups have been incorporated into existing or new collections. Fiocruz collections act as a testimony to the biological diversity of ecosystems often already completely degraded. They reflect the arrangements of biological communities under the evolutionary pressures of that time. These collections support research to identify and confront genetic mutations found in the present, fundamental knowledge for forecasting the dispersion of infectious diseases and developing blocking and therapeutic strategies. Additionally, because collections have been primarily formed from specimens collected during disease outbreaks and epidemiological investigations, Fiocruz collections represent the biological diversity of human-modified habitats. This is another aspect in which Fiocruz collections differ from other repositories, since biodiversity researchers generally prefer to conduct biological surveys in habitats as pristine as possible. Lastly, they are an essential part of the infrastructure for conservation, deposit, supply, characterisation and taxonomic identification of biological material for the development of scientific research, technology and innovation, as well as epidemiological surveillance, in accordance with existing national and international norms and laws (daSilva and Sá 2016).

## Institutional recognition, development and synergism

Fiocruz was one of the first institutions in Brazil to develop institutional policies for the biological collections under its responsibility. The movement of organisation and institutional recognition of the Fiocruz biological collections started in 2006, with the creation of the Fiocruz Permanent Forum of Biological Collections, composed of representatives from the different collections. This Forum was subsequently transformed to Fiocruz Biological Collections Technical Chamber due to the importance of those collections to the institution. The first output from the Forum was an institutional guide that settled the policies and strategies for the development of biological collections. In this context, the first line of action was to evaluate the current situation of these collections. Four main criteria for the institutional recognition of the biological collections were then defined: 1) to conduct activities such as preservation, deposits, distribution and taxonomic identification of microorganisms, besides scientific consultancy and training; 2) to have a curator; 3) to conduct registration of procedures and maintenance of documentation; and 4) to possess at least minimal human resource and infrastructure (daSilva et al. 2011).

The evaluation demonstrated the need to harmonise procedures and protocols. Therefore, the flow for institutional recognition of the collections, including the evaluation procedure and the responsibilities of the curator and substitute were defined and formalised by the Manual of Organization of Fiocruz Biological Collections and the policy for the access of data and information on the Biological Collections was established (daSilva et al. 2011). This document is constantly updated and is available, with other important documents, in the Fiocruz website dedicated to the Biological Collections (https://portal.fiocruz.br/en/biological-collections).

There is great institutional effort to computerise all biological collections, starting with the implementation in all microbiological collections of the Information System on Collections of Biotechnological Interest (SICol). With this experience, Fiocruz initiated the development of its own biological collection management system (Fiocol) aimed at the zoological collections and later decided that this system will be developed for all Fiocruz biological collections, replacing microSICol in the microbiological collections. Additionally, all Fiocruz microbiological, zoological, botanical and archaeopaleontological collections have their own webpages and are integrated in speciesLink and published in the Information System on Brazilian Biodiversity (SiBBr) and Global Biodiversity Information Facility (GBIF). Soon these biological collections will gradually become registered in the Global Genome Biodiversity Network (GGBN), through which they will provide genomic metadata and material discovery for access. Regarding the microbiological collections, for many years they have been registered with the World Federation of Culture Collection (WFCC). The botanical collection is now listed in Index Herbariorum.

The institution has also invested much effort to implement a quality management system, in accordance to the standard NBR ISO/IEC 17025 for testing and calibration laboratories and to the Best Practice Guidelines for Biological Resource Centers of the Organization for Economic Cooperation and Development (OECD). Fiocruz, in 2011, was the first Brazilian institution to have a microbial collection with accredited testing by the NBR ISO/IEC 17025. In this context, Fiocruz actively participates in the Special Biotechnology Study Committee ABNT/CEE-27, a mirror committee of the international technical committee ISO/TC 276 - Biotechnology. This Committee aims at standardisation in the field of Biotechnology comprising biobanks, which include microbial, animal and plant collections. The first result of this international committee was the publication of ISO 20387: 2018 Biotechnology - Biobanking - General requirements for biobanking, which can be implemented by Fiocruz's biological collections.

Currently, an evaluation of all Biological Collections is conducted annually, through the Quality Management Monitoring System (SAGeQ), an electronic system developed by Fiocruz for its laboratories. In the case of Biological Collections, more specific questions related to the activities of the collections were added. Thus it is possible to assess their productivity and the difficulties they face, in addition to the implementation of the quality management system, allowing the identification of potential improvements and areas that may need institutional action.

Still on institutional efforts, since 2010, there has been an outsourcing contract for part of the Biological Collections, which is being expanded to other collections within Fiocruz. As for financing, from 2012 to 2015, Fiocruz had a specific budgetary action for the Biological Collection (20AQ) from the federal government. Unfortunately, this action was suspended and after many requests from the curators, from 2018 forward, the Institution began to provide a specific resource from its own budget.

## Taxonomic coverage

Currently, thirty-three biological collections are institutionally recognised, all of which have institutional support for their maintenance and safeguarding. They are divided into five categories: microbiological, zoological, botanical, histopathological and archaeopaleontological collections. The specimens represented cover part of the genetic diversity of bacteria, fungi, protozoa, helminths, arthropods, molluscs of medical and environmental importance, exsiccates of medicinal plants, human and animal histopathological samples, samples from archaeological and paleontological sites, as well as recent faeces from vertebrates (Table 1). In addition to these taxonomic groups, if it were not for the pandemic caused by COVID-19, viruses would be included. Institutional recognition of the new virus collection (Collection of Viruses and Monoclonal Antibodies—CVAM), which is already structured and receiving deposits, was expected in early 2020. Ironically, the institutional recognition of Fiocruz's first virus collection was postponed because of a virus, SARS-CoV-2.

A few institutions in Brazil have the primary mission of serving as repositories of biodiversity (see Peixoto et al. 2006), amongst them: Museu Nacional (MNRJ, Rio de Janeiro, which lost part of its collections in 2018), Museu Paraense Emílio Goeldi (MPEG, Pará), Museu de História Natural do Capão da Imbuia (MHNCI, Paraná) and Museu de Biologia Prof. Mello Leitão (MBPML [currently Instituto Nacional da Mata Atlântica]) host zoological and botanical collections; Instituto Nacional de Pesquisas da Amazônia (INPA, Amazonas) host zoological, botanical and microbiological collections; Museu de Zoologia da Universidade de São Paulo (MZUSP, São Paulo) hosts zoological collections, including fossils; and Jardim Botânico do Rio de Janeiro (JBRJ, Rio de Janeiro) hosts botanical, fungi and insect collections. In general, collections from MNRJ, MZUSP and JBRJ cover different Brazilian biomes, whereas those from MPEG and INPA are focused on the Amazon region; and those from MHNCI and MBPML are more geographically restricted to the Atlantic Forest of Paraná and Espírito Santo, respectively. In this context, we can assume that those institutions that are primarily devoted to serve as repositories of biodiversity in Brazil have most of their collections focused on the diversity of animals and plants, with fungi and microorganisms under-represented.

Besides these institutions, many other institutions keep biological collections, mostly federal and state universities. Most of these collections represent the local fauna, flora and microbiota and do not have institutional recognition, putting them at great risk in the medium and long term. On the other hand, a few other institutions of education and agroforestry are playing a key role as nationwide repositories. Amongst them, Universidade Federal do Paraná (UFPR), which houses an important entomological collection, as well as other vertebrate and invertebrate collections, besides microbiological collections; State University of Campinas (Unicamp), which hosts the Brazilian Collection of Environmental and Industrial Microorganisms (CBMAI); Federal University of Pernambuco (UFPR), which hosts the Micoteca URM; and the Herbarium of the State University of Feira de Santana (HUEFS) that has the largest collection of Angiosperms of north-eastern Brazil.

The Brazilian Agricultural Research Corporation (Embrapa) houses collections of germoplasm, fungi and microorganisms of agricultural and industrial importance. Against this background, we can assume that Fiocruz collections play a complementary role in representing biodiversity in Brazil (Table 2) inasmuch as: 1) no other Brazilian institution whose focus is not to serve as repository of biodiversity keeps as many collections as Fiocruz; and 2) its microbiological, botanical and zoological collections represent animals, plants, fungi and microorganisms of medical importance, including synanthropic and alien species that are not represented or under-represented in other collections; and 3) collections with historical series that individually correlate etiological agents, hosts, tissues and their pathologies.

## Perspectives

Based on the extensive experience with Fiocruz microbial collections, the Institution is dedicated to the construction of the Biological Resources Center for Health (Health BRC). This centre will focus on microorganisms related to tropical diseases of Latin America, including neglected diseases, as well as microorganisms with taxonomical and biotechnological interest. The Fiocruz Health-BRC will offer certified products and services to the scientific community, industry and the Brazilian Unified Health System (SUS), in order to provide sustainability for biotechnological innovations in health, as well as the conservation of the microbial diversity in the country (daSilva et al. 2011). The Fiocruz Health-BRC will be part of the Brazilian Network of BRCs (BRC-Br Network), which is being structured within the scope of the Ministry of Science, Technology, Innovation and Communication (MCTIC) with funding from the National Fund for Scientific and Technological Development (FNDCT/MCTIC).

Consideration should also be given to efforts to incorporate new biological collections that will, in the future, add new taxonomic groups, such as viruses.

Fiocruz plans to expand access to the collections. In addition to consultation of the physical specimens held by these biological collections, three-dimensional scans of zoological and histopathological specimens will be available very soon. Thanks to an institutional project funded by the Brazilian Development Bank (BNDES), the specimens images will be available online, along with other identifying information, allowing researchers to access these specimens remotely, even offering the ability to measure body parts.

As the Fiocruz Biological Collections are registered in the national systems that contribute to the GBIF, it is expected these Collections will largely meet the requirements being built by the TDWG Collection Descriptions Data Standard Task Group, which is in the process of developing a new formal standard for the technical description of natural science collections (https://www.tdwg.org/community/cd/). This functionality is particularly important when a full catalogue of specimens is not available and for more complex biological collections that deal with ecological interactions, such as parasitic species and infectious agents. In these cases, the transmission and infection processes involve several species of hosts and/or vectors. The difficulty of tracking and linking datasets and metadata of pathogens, host species or substrates from which they were isolated, restricts studies into the ecology of diseases, dispersion and risks.

## Figures and Tables

**Table 1.** Information on the Fiocruz Biological Collections.

**Biological Collections of Fiocruz**	**Taxonomic scope**	**Size**	**Method of preservation**	**Links to the catalogue**
Yersinia pestis Collection (Fiocruz/CYP)	Bacteria	917 strains	Cryopreservation at -80°C; on agar at 8°C	http://cyp.fiocruz.br/
Collection of Campylobacter (Fiocruz/CCAMP)	Bacteria	1,677 strains	Cryopreservation in liquid nitrogen; at -70°C	http://ccamp.fiocruz.br/
Leptospira Collection (Fiocruz/CLEP)	Bacteria	344 strains	Cryopreservation in liquid nitrogen; at -80°C; on semi-solid agar at room temperature	http://clep.fiocruz.br/
Listeria Collection (Fiocruz/CLIST)	Bacteria	1,464 strains	On solid and semi-solid buffered agar at 4°C	http://clist.fiocruz.br/
Culture Collection of Bacillus and Related Genera (Fiocruz/CCGB)	Bacteria	1,427 strains	Cryopreservation in liquid nitrogen; lyophilisation	http://ccgb.fiocruz.br/
Culture Collection of Hospital-Acquired Bacteria (Fiocruz/CCBH)	Bacteria	600 strains	Cryopreservation in liquid nitrogen; liophilisation	http://ccbh.fiocruz.br/
Collection of Reference Bacteria on Health Surveillance (Fiocruz/CBRVS)	Bacteria	721 strains	Cryopreservation at -80°C; lyophilisation	http://cbrvs.fiocruz.br/
Collection of Bacteria from Amazon (Fiocruz/CBAM)	Bacteria	716 strains	Lyophilisation; under refrigeration at -40°C; culture on stock agar at room temperature	http://cbam.fiocruz.br/
Collection of Bacteria from Environment and Health (Fiocruz/CBAS)	Bacteria	1,351 strains	Cryopreservation in liquid nitrogen and at -80°C	http://cbas.fiocruz.br/
Collection of Reference Fungi on Health Surveillance (Fiocruz/CFRVS)	Fungi	437 strains	Cryopreservation in liquid nitrogen and at -80°C; lyophilisation	http://cfrvs.fiocruz.br/
Collection of Pathogenic Fungi (Fiocruz/CFP)	Fungi	324 strains	Cryopreservation in liquid nitrogen and at -70°C; lyophilisation	http://cfp.fiocruz.br/
Collection of Fungi from Amazon (Fiocruz/CFAM)	Fungi	1,245 strains	Lyophilisation; under refrigeration at -40°C; culture under water (Castellani) at room temperature	http://cfam.fiocruz.br/
Collection of Culture of Filamentous Fungi (Fiocruz/CCFF)	Fungi	1,188 strains	Lyophilisation; culture under mineral oil at room temperature	http://ccff.fiocruz.br/
Collection of Leishmania (Fiocruz/CLIOC)	Protozoa	1,657 strains	Cryopreservation in liquid nitrogen	http://clioc.fiocruz.br/
Collection of Trypanosoma from Wild and Domestic Mammals and Vectors (Fiocruz/COLTRYP)	Protozoa	883 strains	Cryopreservation in liquid nitrogen	http://coltryp.fiocruz.br/
Protozoa Collection (Fiocruz/COLPROT)	Protozoa	524 strains	Cryopreservation in liquid nitrogen	http://colprot.fiocruz.br/
Collection of Apterous Arthropod Vector of Community Health Importance (Fiocruz/CAVAISC)	Insects and Arachnids (Acari)	14,611 lots and 2051 specimens	Mounted on entomological pin; mounted on slide; in 70% ethanol; in 70% ethanol-glycerin solution	http://cavaisc.fiocruz.br/
Ceratopogonidae Collection (Fiocruz/CCER)	Insects	10,377 specimens	Mounted on slide; in 70% ethanol; Kahle-Dietrich solution	http://ccer.fiocruz.br/
Simuliidae Collection of the Oswaldo Cruz Institute (Fiocruz/CSIOC)	Insects	50,000 lots	Mounted on slide; in 70% ethanol; in glycerin; in Carnoy's solution	http://csioc.fiocruz.br/
Culicidae Collection (Fiocruz/CCULI)	Insects	6,989 specimens	Mounted on entomological pin; mounted on slide	http://cculi.fiocruz.br/
Collection of Neotropical Mosquitos (Fiocruz/CMN)	Insects	63,469 specimens	Mounted on entomological pin; mounted on slide	http://cmn.fiocruz.br/
Collection of Phlebotominae (Fiocruz/COLFLEB)	Insects	93,000 specimens	Mounted on slide	http://colfleb.fiocruz.br/
Triatomines Collection of the Oswaldo Cruz Institute (Fiocruz/CTIOC)	Insects	11,590 specimens	Mounted on entomological pin	http://ctioc.fiocruz.br/
Collection of Chagas Disease Vectors (Fiocruz/COLVEC)	Insects	13,295 specimens	Mounted on entomological pin	http://colvec.fiocruz.br/
Entomological Collection of the Oswaldo Cruz Institute (Fiocruz/CEIOC)	Insects	1,000,000 specimens	Mounted on entomological pin; mounted on slide; in 70% ethanol	http://ceioc.fiocruz.br/
Helminthological Collection of the Oswaldo Cruz Institute (Fiocruz/CHIOC)	Helminths	39,633 specimens	Mounted on slide; in 70% ethanol; in 10% formalin; in ethanol-formalin-acetic acid; under refrigeration at -30°C	http://chioc.fiocruz.br/
Mollusca Collection of the Oswaldo Cruz Institute (Fiocruz/CMIOC)	Molluscs	12,317 lots	Shell preserved dry; body in Railliet & Henry solution	http://cmioc.fiocruz.br/
Collection of Medical Malacology (Fiocruz/CMM)	Molluscs	16,765 specimens	Shell preserved dry; body in Railliet & Henry solution; small fragment of cephalopodal region cryopreserved at -70°C	http://cmm.fiocruz.br/
Botanical Collection of Medicinal Plants (Fiocruz/CBPM)	Plants	1,732 exsiccatae	Herborised then dried and mounted on exsiccates	http://cbpm.fiocruz.br/
Yellow Fever Collection (Fiocruz/CFA)	Histopathological samples	500 slides 500 blocks 500 backup material	Mounted on slide; paraffin block; in 10% formalin	http://museudapatologia.ioc.fiocruz.br/index.php/en/museum-pathology/cfa-history.html
Anatomical Pathology Section Collection (Fiocruz/CSAP)	Histopathological samples	40,000 slides 854 anatomical pieces	Mounted on slide; In 10% formalin	http://museudapatologia.ioc.fiocruz.br/index.php/en/museum-pathology/apsc.html
Pathology Department Collection (Fiocruz/CDEPAT)	Histopathological samples	133,280 blocks 799,680 slides	Mounted on slideparaffin block; in Carson Modified Millonig formalin	http://museudapatologia.ioc.fiocruz.br/index.php/en/museum-pathology/cdepat.html
Paleoparasitology and Recent Animal Faeces Collection (Fiocruz/CPFERA)	Bioarchaelogical materials, recent animals faeces	8,584 samples 323 lots	Dried; in Railliet & Henry solution; in 70% ethanol; in natural at -80°C	http://cpfera.fiocruz.br/

**Table 2.** Taxonomic representativeness in the biological collections and host institutions in Brazil.

Biological Collections	MNRJ	MPEG	INPA	MZUSP	JBRJ	Embrapa	Fiocruz
Plants	X	X	X		X		
Medicinal Plants							X
Phytopathological Material						X	
Germoplasm						X	
Insects	X			X	X		X
Insects of Medical Importance							X
Fossils	X			X			
Vertebrates	X	X	X	X			
Invertebrates	X	X	X	X			X
Invertebrates of Medical Importance							X
Fungi					X		X
Fungi of Medical Importance							X
Fungi of Agricultural Importance						X	
Fungi of Environmental Importance							X
Microorganisms							X
Microorganisms of Medical Importance							X
Microorganisms of Agricultural Importance						X	
Microorganisms of Industrial Importance						X	X
Microorganisms of Environmental Importance							X
Histopathological Material							X
Archeopaleontological Material							X
Institutions and acronyms: Museu Nacional (MNRJ); Museu Paraense Emílio Goeldi (MPEG); Instituto Nacional de Pesquisas da Amazônia (INPA); Museu de Zoologia da Universidade de São Paulo (MZUSP); Jardim Botânico do Rio de Janeiro (JBRJ); Empresa Brasileira de Pesquisa Agropecuária (Embrapa); Fundação Oswaldo Cruz (Fiocruz).							
